# Validation and Comparison of Reference Genes for qPCR Normalization of Celery (*Apium graveolens*) at Different Development Stages

**DOI:** 10.3389/fpls.2016.00313

**Published:** 2016-03-17

**Authors:** Meng-Yao Li, Feng Wang, Qian Jiang, Guan-Long Wang, Chang Tian, Ai-Sheng Xiong

**Affiliations:** State Key Laboratory of Crop Genetics and Germplasm Enhancement, College of Horticulture, Nanjing Agricultural UniversityNanjing, China

**Keywords:** celery, reference gene, qPCR, gene expression, developmental stage

## Abstract

A suitable reference gene is an important prerequisite for guarantying accurate and reliable results in qPCR analysis. Celery is one of the representative vegetable in Apiaceae and is widely cultivated and consumed in the world. However, no reports have been previously published concerning reference genes in celery. In this study, the expression stabilities of nine candidate reference genes in leaf blade and petiole at different development stages were evaluated using three statistics algorithms geNorm, NormFinder, and BestKeeper. Our results showed that *TUB-B, TUB-A*, and *UBC* were the most reference genes among all tested samples. *GAPDH* represented the maximum stability for most individual sample, while the *UBQ* displayed the minimum stability. To further validate the stability of reference genes, the expression pattern of *AgAP2-2* was calculated by using the selected genes for normalization. In addition, the expression patterns of several development-related genes were studied using the selected reference gene. Our results will be beneficial for further studies on gene transcription in celery.

## Introduction

Celery is widely cultivated as a vegetable crop in the world, and is rich in flavonoids, carotenoids, carbohydrate, and fibrin. During the plant development process, the physiological and morphological characteristics of celery have significant changes, which will affect the eating quality of leaf blade and petiole. In the last few years, high throughput sequencing technology has been widely used in celery research, for exploration gene transcriptional mechanism and regulation networks (Jiang et al., 2014a,b; Li et al., 2014a,b; Jia et al., 2015). These studies have identified many key genes that involved in lignin biosynthetic pathway during celery development stages (Jia et al., 2015). Another study indicated that some genes were associated with apigenin biosynthesis during celery leaf development (Yan et al., 2014). However, more regulatory networks during plant development are still waiting to be studied.

Gene expression pattern reflects the tendency of gene expression regulation, and provides a novel insight into understand the biological functions of genes. qPCR is a reliable and rapid method to evaluate the expression level of target gene, especially has very sensitive detection ability for some low copy mRNAs (Heid et al., 1996; Bustin, 2000; Mackay, 2004). To exclude the errors in mRNA extraction quality, reverse amplification efficiency, and qPCR procedures, the reference genes are needed for data correction and standardization (Radonić et al., 2004; Dheda et al., 2005). An ideal reference gene should have a constant expression in various tissues and different experimental conditions (Dheda et al., 2004). However, to date, no absolute reference gene has been identified in plants or animals (Volkov, 2003; Derveaux et al., 2010). Some studies directly selected the common reference genes such as *ACTIN, GAPDH*, and *TUB* to normalize the target genes without evaluating the expression stability. However, these reference genes have significant differences under different experimental conditions (Kim et al., 2003; Yan et al., 2012). The unstable expression of reference genes may cause the deviation of final result. Other researches pointed that two or more reference genes should be needed to normalize (Vandesompele et al., 2002; Schmid et al., 2003). Some valid statistical software have been developed, such as geNorm (Vandesompele et al., 2002), Bestkeeper (Andersen, 2004), NormFinder (Pfaffl et al., 2004), to evaluate the stability of the candidate reference genes under specific experimental conditions.

Currently, several reliable reference genes have been reported in plants, and the stability of reference genes in different plant species are not completely consistent (Czechowski et al., 2005; Jiang et al., 2014a; Tian et al., 2015). *ACT7* and *PP2A* genes displayed the maximum stability under abiotic stress conditions in *Oenanthe javanica* (Bl.) (Jiang et al., 2014a), *ACTIN* and *TUB* were the most stable genes in carrot (Tian et al., 2015). Moreover, the reference genes under different experimental conditions are also not the same. In the study of rice, *eIF-4*α and *ACT1* were the most suitable reference genes during seed development (Li et al., 2010), *UBQ5* and *eEF-1*α were most stable across all the tissue samples (Jain et al., 2006), while the *18S* rRNA was the most reliable reference gene under various growth stages of etiolated seedlings and different cultivars (Kim et al., 2003). However, none of reference gene in celery has been reported. Hence, identification of suitable reference genes in various tissues and at different development stages will be required, which will greatly contribute accurate and reliable analysis of gene expression.

To accurately normalize the target gene expression in celery tissues and development stages, nine candidate reference genes were selected and their expression stability was evaluated. The target gene *AgAP2-2*, a gene encoding an AP2/ERF transcription factor, was used to validate the selection of reference gene. In addition, the expression patterns of development-related genes were also analyzed using the selected reference gene. This study aims to identify the most suitable reference genes that will provide a more accurate and reliable expression analysis of other celery genes among various tissues and development stages.

## Materials and methods

### Plant materials and growth conditions

**Figure 1:**
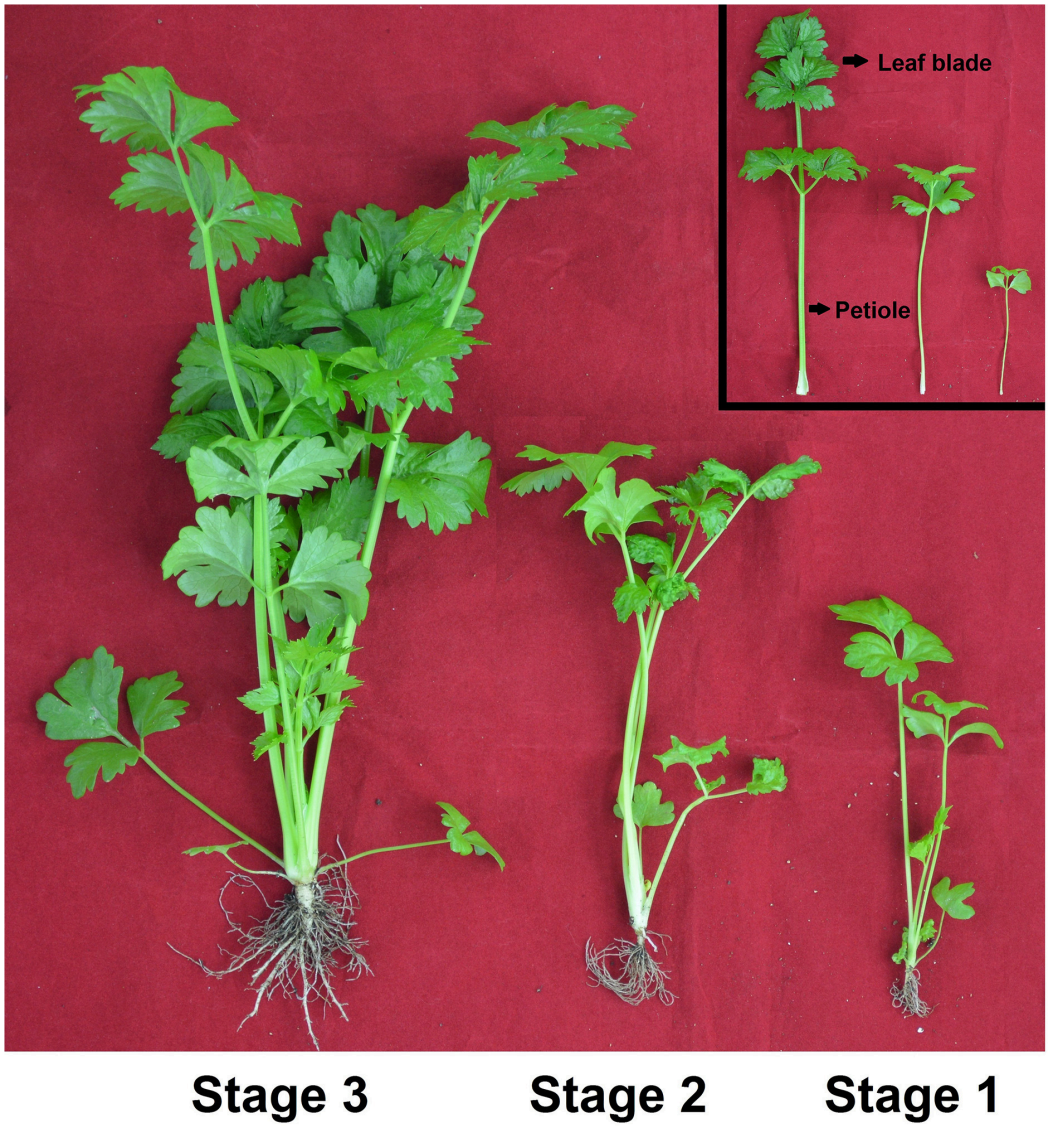
**Growth status of celery at three developmental stages**. The leaf blades and petioles at different developmental stages were presented, respectively. Stage 1, 35 days after sowing; Stage 2, 50 days after sowing; Stage 3, 60 days after sowing.

The seeds of celery (*Apium graveolens* L. cv. Ventura) were cultivated in a controlled-environment growth chamber in Nanjing Agricultural University, China (32°02′N, 118°50′E). All plants were grown under a photoperiod of 16 h with 300 μ mol m^−2^s^−1^ light intensity at 25°C and 8 h dark condition at 16°C. The relative humidity varied from 60 to 65%. Three development stages of celery were evaluated, and the height of the plant at Stage 1 was 10 cm (35 d), the height of the plant at Stages 2 was 20 cm (50 d), and the height of the plant at Stages 3 was 30 cm (65 d; Figure 1). Three biological replicate samples of celery leaf blade and petiole at each developmental stage were collected, then immediately frozen in liquid nitrogen and stored at −80°C until use.

### RNA isolation and cDNA synthesis

Total RNA was extracted using the total RNA kit (Tiangen, Beijing, China) and then treated with RNase-free DNase I (Takara, Dalian, China) to eliminate genomic DNA contamination. The quantity and quality of RNA samples were measured by agarose gel electrophoresis and the use of a Nanodrop ND 1000 spectrophotometer (Nanodrop Technologies Inc., Delaware, USA). Only the samples with an A_260_/A_280_ ratio of 1.8–2.2 and an A_260_/A_230_ ratio >1.8 were used for further analysis. Total RNA (1.0 μg) was reverse-transcribed into cDNA using a PrimeScript RT reagent kit (Takara, Dalian, China). The cDNA was efficacy dilutions (10X, 10^2^X, 10^3^X, 10^4^X, 10^5^X dilution) for detecting the amplification efficiency (*E*) and correlation coefficient (*R*^2^), and 18-fold dilution for qPCR experiments.

### Selection of candidate reference genes and primer design

Nine typical candidate reference genes of celery, *ACTIN, EF-1*α, *GAPDH, RAP2, TBP, TUB-A, UBC, TUB-B*, and *UBQ*, were selected for qPCR analysis in this study. *Arabidopsis* reference genes were downloaded from the TAIR database (http://www.arabidopsis.org) and used as query sequences to retrieve the homologs genes in celery. Based on the *A*. *graveolens* transcriptome sequencing data built by our group (Li et al., 2014a; Jia et al., 2015), nine potential genes were cloned. We have submitted all the nucleotide sequences to GenBank, and the corresponding accession numbers were KU234487 (*ACTIN*), KU234488 (*EF-1*α), KU234489 (*GAPDH*), KU234490 (*RAP2*), KU234491 (*TBP*), KU234492 (*TUB-A*), KU234493 (*UBC*), KU234494 (*TUB-B*), and KU234495 (*UBQ*). The primers used for qPCR were designed by Primer Premier 6 using a standard set of design criteria (annealing temperatures 58–60°C, primer lengths 18–26 bp, GC content between 40 and 60%, and the PCR product between 60 and 150 bp; Udvardi et al., 2008). Primer sequences and amplicon characteristics are listed in Table 1.

**Table 1:** **Descriptions of candidate reference genes and primer sequences for qPCR**.

**Gene**	***Arabidopsis* homolog gene**	**Primer sequence (5′–3′)**	**Amplicon size (bp)**	**Annealing *Tm* (°C)**	**Melting *Tm* (°C)**
*ACTIN*	AT5G09810	AGAAGTCCTGTTCCAGCCGTCTT/CGAACCACCACTGAGCACTATGTT	136	59.6	82.0
*EF-1α*	AT1G07940	GTCACCAGGAAGTGCCTCTGTAAG/TGTACCTGTCGGACGAGTTGAGA	136	59.2	84.0
*GAPDH*	AT1G42970	CAAGGACTGGAGAGGTGGAAGAG/GTGAGGTCAACAACTGAGACATCC	159	57.9	83.5
*RAP2*	AT1G53910	GCTTATGATGCTGAGGCAAGGAGA/TGGTACAGAGCCGAACGAGAGT	155	59.2	83.5
*TBP*	AT1G55520	CTGGAGCAAAGAGCGAACAACAAT/GCAAGACCTTCAAGCCTGATGG	157	57.9	82.0
*TUB-A*	AT4G14960	CCTCACCACAGGTCTCAACTTCAG/GGTGTAGGTTGGACGCTCAATGT	158	59.3	84.0
*UBC*	AT1G16890	AGGCTTGAGATTCGCTGTCTGTAA/TATTCCTGGAGCTGGCTCACTGA	158	59.3	81.5
*TUB-B*	AT5G23860	TGGTGGCACTGGATCTGGTATGG/ACTTTCGGAGGAGGGAAGACTGAA	105	59.5	80.0
*UBQ*	AT4G05320	GAAGATGGAAGAACTCTCGCAGAT/CGGTCAATGGTATCAGTTGGTTCA	152	57.6	80.5

### qPCR and statistical analysis

The qPCR reactions were performed in a 96 well plate using the MyiQ Single Color Real-Time PCR Detection System (Bio-rad, Hercules, CA, USA). Each 20 μL PCR reactions contained 2.0 μL of diluted cDNA, 0.4 μL of each primer (10 mM), 10 μL of SYBR Green I mix (Takara, Dalian, China), and 7.2 μL of ddH_2_O. The PCR conditions were as follows: at 95°C for 30 s for pre-denaturation, 40 cycles of 95°C for 5 s for denaturation, and 60°C for 30 s for annealing and extension. A melting curve (65–95°C, at increments of 0.5°C) was generated to verify the specificity of primer amplification. Each PCR reaction was repeated three times, and three biological replicates were analyzed. In Addition, each assay contained a standard curve of different dilutions of the template and a no-template control. Amplification efficiency of each primer pair was calculated (%*E* = (−1+10^[−1∕slope]^) × 100%) and correlation coefficient (*R*^2^) was tested.

Expression level of nine genes in each reaction was determined by the cycle threshold Cq (the cycle at which a threshold fluorescence was obtained). The original data was presented in the Table S1. Three different Microsoft Excel-based softwares, geNorm (Vandesompele et al., 2002), NormFinder (Andersen, 2004), and BestKeeper (Pfaffl et al., 2004), were used to determine the best reference genes. These raw data can be directly used for BestKeeper program, but for geNorm and NormFinder, Cq-values were converted into relative quantity values by the formula 2^−ΔCq^, ΔCq = the corresponding Cq-value – minimum Cq.

1. geNorm. In geNorm, the calculation principle relies on the expression ratio of two ideal internal control genes is identical in all samples. The gene expression stability measure *M* is calculated as the level of pairwise variation for that gene compared with all other tested reference genes. geNorm identify these genes by progressively eliminating less stable genes from the analysis, and the reference gene with the lowest pairwise variation is the most stable. Besides, optimal number of multiple reference genes was determined by pairwise variation (*V*_*n*__∕__*n*__+1_) between normalization factors (NF_*n*_ and NF_*n*__+1_, *n* ≥ 2) in geNorm.
2. NormFinder. The NormFinder program ranks all candidate reference genes on the basis of intragroup and intergroup expression variations, and then combines them into a stability value for each candidate reference gene. This program can avoid the misinterpretations which caused by artificial selection of co-regulated genes. The reference gene with the lowest stability value is the most stable.
3. BestKeeper. BestKeeper ranks the candidates' stability based on the standard deviation (*SD*) and the coefficient of variation (*CV*) with the Cq-values of all genes. Candidate reference gene with the lowest *SD*- and *CV*-values is considered as the most stable gene.

### Selection and expression analysis of development related genes

The transcriptome sequencing of three celery development stages were finished by our group (Li et al., 2014a; Jia et al., 2015). Base on the annotation of celery genes, some of the genes were related to plant growth and development. Transcript abundances were estimated by calculating read density as “reads per kilobase of exon model per million mapped reads” (RPKM; Mortazavi et al., 2008). The expression clusters of genes were analyzed by using Cluster (http://bonsai.hgc.jp/~mdehoon/software/cluster/software.htm), and the heatmap was drawn using Tree View (http://jtreeview.sourceforge.net/).

qPCR was also performed to analyze the gene expression. Each reaction had three technical and biological repeats. The relative gene expression levels were calculated with the 2^−ΔΔCT^ method (Pfaffl, 2001). The gene-specific primers are shown in Table S2.

## Results

### Amplification specificity and efficiency of candidate reference genes

**Figure 2:**
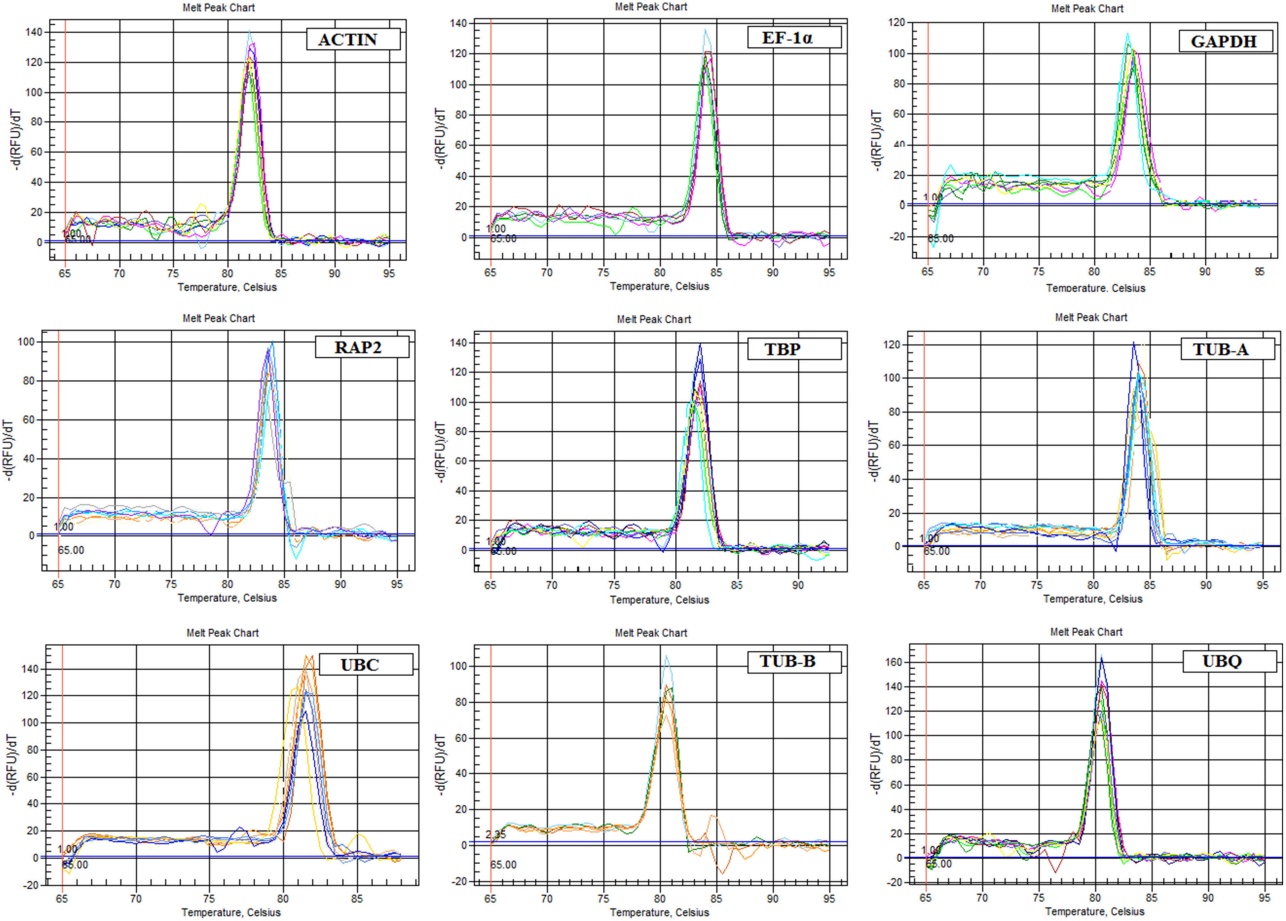
**Melting curves generated for nine candidate reference genes by qPCR**.

The specific primers of nine candidate reference genes were designed for qPCR, with the amplicon length ranging from 105 to 159 bp. A single peak in the melting curve showed the expected amplification effect (Figure 2). The correlation coefficients (*R*^2^) and PCR amplification efficiencies of nine genes in leaf blade and petiole were calculated, respectively, the results met the standard (*R*^2^ > 0.99, 90 < *E*% < 110; Ramakers et al., 2003; Figures S1, S2).

**Figure 3:**
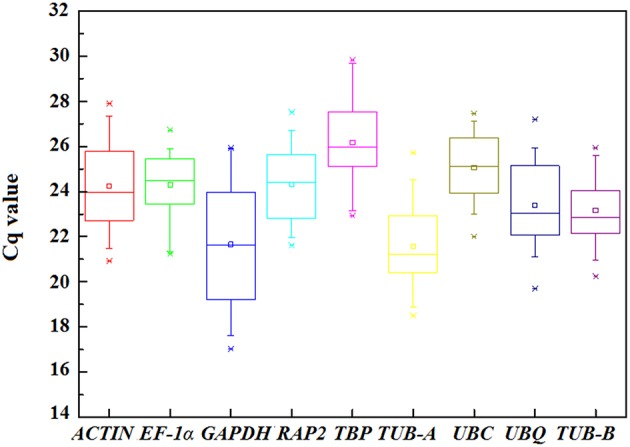
**Distribution of the Cq-values of the nine candidate reference genes across all samples in qPCR analysis**. The straight line crossing the box depicts median and the inside box represent mean. The outside box indicates the 25th and 75th percentiles. Whiskers represent the 5th and 95th percentiles. Asterisk represents outlier.

The Cq-values in qPCR provided an overview of the gene expression levels of nine candidate reference genes in test samples. The transcript abundant for each gene has significant difference and the raw data were listed in Table S2. In general, the Cq-values between 18 and 30 are considered to be appropriate and effective data (Czechowski et al., 2005; Derveaux et al., 2010; Jiang et al., 2014a). In this study, all the Cq-values were between 17.03 and 29.84, and the mean Cq-values of the genes ranged from 21.57 for *TUB-A* to 26.18 for *TBP* (Figure 3 and Table S3).

High Cq-value indicates the low expression levels, conversely mean the high expression. Among nine genes, *GAPHD* and *TUB-A* showed high expression with low Cq-values, whereas *UBC* and *TBP* showed low expression. The significant variation in gene expression indicated screening the appropriate reference gene should measure the stability.

### Stability of candidate reference genes under different development stages and tissues

To select the most suitable reference gene, three methods (geNorm, NormFinder, and BestKeeper) were used to analyze the stability of each reference gene. The stability ranking of candidate reference genes in six individual samples were calculated, respectively (Table 2). In addition, we divided these six samples into three groups: “Leaf blade,” “Petiole,” and “Total.” For complexity of the groups, the ranks of the nine genes were calculated again and the results were shown in Table 3.

**Table 2:** **The stability ranking of candidate reference genes in individual tissue sample by geNorm, NormFinder, and BestKeeper**.

**Treatments**	**Rank**	**geNorm**		**NormFinder**		**BestKeeper**		
		**Gene**	**Stability**	**Gene**	**Stability**	**Gene**	***SD***	***CV***
Stage 1, Leaf blade	1	*GAPDH*	0.31	*GAPDH*	0.014	*TUB-A*	0.34	1.62
	2	*TBP*	0.31	*UBC*	0.014	*TUB-B*	0.36	1.61
	3	*UBC*	0.40	*TBP*	0.017	*UBC*	0.37	1.47
	4	*TUB-A*	0.46	*TUB-B*	0.061	*GAPDH*	0.38	1.78
	5	*TUB-B*	0.57	*TUB-A*	0.070	*RAP2*	0.48	2.10
	6	*RAP2*	0.62	*RAP2*	0.143	*TBP*	0.55	2.10
	7	*EF-1α*	0.72	*ACTIN*	0.183	*ACTIN*	0.65	2.87
	8	*ACTIN*	0.80	*EF-1α*	0.280	*EF-1α*	0.78	3.44
	9	*UBQ*	0.92	*UBQ*	0.312	*UBQ*	0.89	4.04
Stage 2, Leaf blade	1	*GAPDH*	0.36	*ACTIN*	0.009	*RAP2*	0.38	1.51
	2	*UBC*	0.36	*TBP*	0.013	*TUB-B*	0.38	1.51
	3	*TUB-B*	0.41	*TUB-B*	0.020	*TBP*	0.55	1.96
	4	*RAP2*	0.52	*GAPDH*	0.021	*ACTIN*	0.58	2.23
	5	*ACTIN*	0.73	*EF-1α*	0.024	*UBC*	0.58	2.21
	6	*TBP*	0.83	*RAP2*	0.031	*GAPDH*	0.61	2.57
	7	*EF-1α*	0.88	*UBC*	0.033	*EF-1α*	0.64	2.55
	8	*UBQ*	0.95	*TUB-A*	0.075	*UBQ*	0.95	4.28
	9	*TUB-A*	1.04	*UBQ*	0.218	*TUB-A*	1.16	5.07
Stage 3, Leaf blade	1	*UBC*	0.33	*TUB-B*	0.005	*UBC*	0.21	0.80
	2	*EF-1α*	0.33	*EF-1α*	0.009	*EF-1α*	0.22	0.85
	3	*GAPDH*	0.51	*GAPDH*	0.013	*GAPDH*	0.41	1.63
	4	*TUB-B*	0.69	*ACTIN*	0.013	*TUB-B*	0.49	1.91
	5	*RAP2*	0.73	*UBC*	0.017	*RAP2*	0.59	2.27
	6	*TUB-A*	0.78	*TUB-A*	0.017	*TUB-A*	0.64	2.69
	7	*ACTIN*	0.84	*TBP*	0.018	*ACTIN*	0.77	2.90
	8	*TBP*	0.91	*RAP2*	0.030	*TBP*	0.92	3.24
	9	*UBQ*	1.00	*UBQ*	0.075	*UBQ*	1.13	4.73
Stage 1, Petiole	1	*UBC*	0.56	*TBP*	0.094	*UBQ*	0.20	0.86
	2	*UBQ*	0.56	*UBQ*	0.114	*TUB-B*	0.42	1.96
	3	*TUB-B*	0.65	*TUB-A*	0.228	*UBC*	0.47	1.98
	4	*GAPDH*	0.69	*RAP2*	0.250	*TBP*	0.78	3.14
	5	*TUB-A*	0.74	*UBC*	0.257	*GAPDH*	0.79	4.28
	6	*TBP*	0.92	*TUB-B*	0.260	*TUB-A*	0.84	4.28
	7	*RAP2*	1.07	*EF-1α*	0.311	*RAP2*	1.01	4.32
	8	*EF-1α*	1.17	*ACTIN*	0.316	*EF-1α*	1.20	5.23
	9	*ACTIN*	1.30	*GAPDH*	0.355	*ACTIN*	1.38	6.02
Stage 2, Petiole	1	*GAPDH*	0.47	*TUB-A*	0.073	*EF-1α*	0.44	1.82
	2	*TUB-B*	0.47	*TUB-B*	0.093	*GAPDH*	0.54	2.89
	3	*TUB-A*	0.56	*GAPDH*	0.097	*TUB-A*	0.55	2.72
	4	*UBC*	0.68	*EF-1α*	0.114	*ACTIN*	0.58	2.46
	5	*EF-1α*	0.80	*UBC*	0.147	*TUB-B*	0.59	2.65
	6	*ACTIN*	0.92	*ACTIN*	0.182	*UBC*	0.74	3.08
	7	*TBP*	1.02	*UBQ*	0.192	*RAP2*	0.76	3.35
	8	*RAP2*	1.10	*TBP*	0.300	*TBP*	0.92	3.82
	9	*UBQ*	1.20	*RAP2*	0.300	*UBQ*	1.27	5.43
Stage 3, Petiole	1	*GAPDH*	0.42	*GAPDH*	0.010	*GAPDH*	0.29	1.33
	2	*TUB-A*	0.42	*EF-1α*	0.015	*TBP*	0.31	1.22
	3	*TUB-B*	0.46	*UBQ*	0.020	*TUB-A*	0.36	1.63
	4	*UBQ*	0.49	*TUB-A*	0.022	*EF-1α*	0.42	1.64
	5	*TBP*	0.54	*TUB-B*	0.039	*UBC*	0.42	1.72
	6	*UBC*	0.58	*RAP2*	0.044	*TUB-B*	0.54	2.34
	7	*EF-1α*	0.68	*TBP*	0.056	*RAP2*	0.65	2.55
	8	*RAP2*	0.80	*UBC*	0.121	*UBQ*	0.71	2.87
	9	*ACTIN*	0.91	*ACTIN*	0.125	*ACTIN*	0.95	3.99

*SD, standard deviation; CV, coefficient of variation*.

**Table 3:** **The stability ranking under “Leaf blade,” “Petiole,” and “Total” by geNorm, NormFinder, and BestKeeper**.

**Group**	**Rank**	**geNorm**		**Normfinder**		**Bestkeeper**		
		**Gene**	**Stability**	**Gene**	**Stability**	**Gene**	***SD***	***CV***
Leaf blade	1	*TUB-B*	0.47	*TUB-B*	0.051	*UBC*	0.78	2.98
	2	*RAP2*	0.47	*TUB-A*	0.054	*TBP*	0.96	3.48
	3	*GAPDH*	0.74	*UBC*	0.059	*UBQ*	1.16	5.11
	4	*TUB-A*	0.81	*TBP*	0.066	*RAP2*	1.21	4.89
	5	*UBC*	0.88	*GAPDH*	0.079	*EF-1α*	1.24	5.11
	6	*EF-1α*	0.95	*RAP2*	0.118	*TUB-B*	1.32	5.36
	7	*TBP*	0.99	*ACTIN*	0.124	*TUB-A*	1.44	6.41
	8	*ACTIN*	1.04	*EF-1α*	0.192	*GAPDH*	1.61	6.84
	9	*UBQ*	1.16	*UBQ*	0.226	*ACTIN*	1.66	6.61
Petiole	1	*TUB-B*	0.73	*UBQ*	0.138	*UBC*	0.75	3.11
	2	*TUB-A*	0.73	*TUB-B*	0.163	*TBP*	0.86	3.45
	3	*UBQ*	0.79	*TUB-A*	0.166	*TUB-B*	0.88	3.94
	4	*UBC*	0.84	*UBC*	0.177	*ACTIN*	1.02	4.37
	5	*GAPDH*	0.94	*EF-1α*	0.201	*EF-1α*	1.08	4.47
	6	*EF-1α*	1.07	*GAPDH*	0.209	*TUB-A*	1.11	5.37
	7	*TBP*	1.17	*ACTIN*	0.232	*UBQ*	1.13	4.78
	8	*RAP2*	1.25	*TBP*	0.248	*RAP2*	1.32	5.53
	9	*ACTIN*	1.34	*RAP2*	0.249	*GAPDH*	1.46	7.41
Total	1	*TUB-B*	0.81	*TUB-B*	0.117	*UBC*	1.13	4.52
	2	*TUB-A*	0.81	*TUB-A*	0.128	*EF-1α*	1.16	4.79
	3	*UBC*	0.87	*UBC*	0.138	*UBQ*	1.22	5.25
	4	*TBP*	1.06	*GAPDH*	0.165	*RAP2*	1.33	5.48
	5	*RAP2*	1.20	*ACTIN*	0.183	*TUB-A*	1.42	6.59
	6	*EF-1α*	1.30	*RAP2*	0.192	*TUB-B*	1.45	6.21
	7	*ACTIN*	1.34	*TBP*	0.199	*TBP*	1.46	5.58
	8	*GAPDH*	1.43	*EF-1α*	0.209	*ACTIN*	1.59	6.56
	9	*UBQ*	1.57	*UBQ*	0.241	*GAPDH*	2.14	9.87

*SD, standard deviation; CV, coefficient of variation*.

In geNorm analysis, the *M*-value was used to represent the average expression stability, the lower the *M*-value indicates a higher stability. In all six samples, the *M*-value of candidate reference genes were less than the default limit of 1.5, but the most suitable reference gene was different in different tissues and development stages. *GAPDH* was the most stable gene at Stages 1 and 2 in leaf blade, and was the most stable gene at Stages 2 and 3 in petiole. *UBC* exhibited relatively stable expression at Stage 3 in leaf blade and Stage 1 in petiole. Under all tissue sets, *TUB-B* was the most stable gene among the nine candidate reference genes in “Leaf blade,” “Petiole,” and “Total,” whereas *UBQ* was the least stable gene with the largest *M*-value in “Leaf blade” and “Total.”

Another method, NormFinder, also classified *TUB-B* as the most stable reference gene with the minimum value of 0.005 at Stage 3 in leaf blade. *GAPDH* showed good stability at Stage 1 in leaf blade and at Stage 3 in petiole. However, *GAPDH* showed the worst stability at Stage 1 in petiole. In three groups, *TUB-B* ranked first in “Leaf blade” and “Total” with the value of 0.051 and 0.117, and ranked second in “Petiole” with the value of 0.163. Moreover, *UBQ* was the most stable gene in “Petiole,” but was the least stable gene in “Leaf blade” and “Total.”

Based on calculations by BestKeeper software, the smaller *SD-* and *CV*-value means the gene is more stable. BestKeeper ranked *TUB-A, RAP2, UBC, UBQ, EF-1*α, *GAPDH*, respectively, as the best reference gene under three development stages in leaf blade and petiole. Although the best reference gene in six individual samples was not the same, *UBC* had the lowest *SD*- and *CV*-values in three groups. That meant *UBC* was more stable than the other genes in three groups “Leaf blade,” “Petiole,” and “Total.” At the same time, we also found that *UBQ* had poor performance according to the ranking by BestKeeper.

### The optimal number of reference genes for normalization in celery

**Figure 4:**
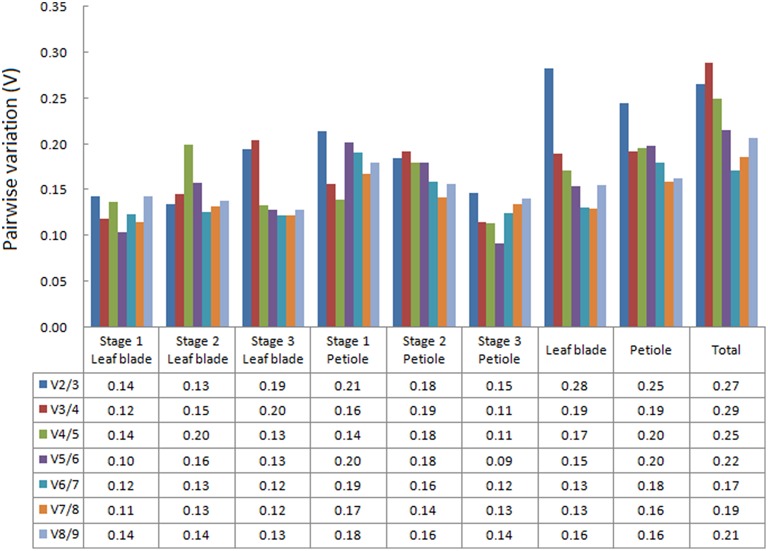
**Determination of the optimal number of reference genes**. Pairwise variation (V_*n*__∕__*n*__+1_) were calculated between the normalization factors (NF_*n*_ and NF_*n*__+1_) in all tested samples. The *V*_*n*__∕__*n*__+1_-values below 0.15 suggested that an additional reference gene is not necessary for normalization.

The geNorm algorithm was used to determine the optimal number of reference genes by evaluating pairwise variation (*V*_*n*__∕__*n*__+1_) between normalization factors (NF_*n*_ and NF_*n*__+1_, *n* ≥ 2). The *V*_*n*__∕__*n*__+1_-value below 0.15 suggested that an additional reference gene is not necessary for normalization (Vandesompele et al., 2002). Among the nine reference genes, the most stable reference genes varied in different samples and groups (Figure 4). For leaf blade development, two reference genes were enough for normalization at Stages 1 and 2, while four reference genes were needed at Stage 3 with the *V*_4∕5_-value dropping to 0.15. At three petiole development stages, four, seven, and two reference genes were needed, respectively. For group “Leaf blade,” five stable reference genes were proposed to be used. When the samples were analyzed as “Petiole” or “Total,” it seems that all the *V*-values were higher than 0.15.

### Validation of the selected reference genes

**Figure 5:**
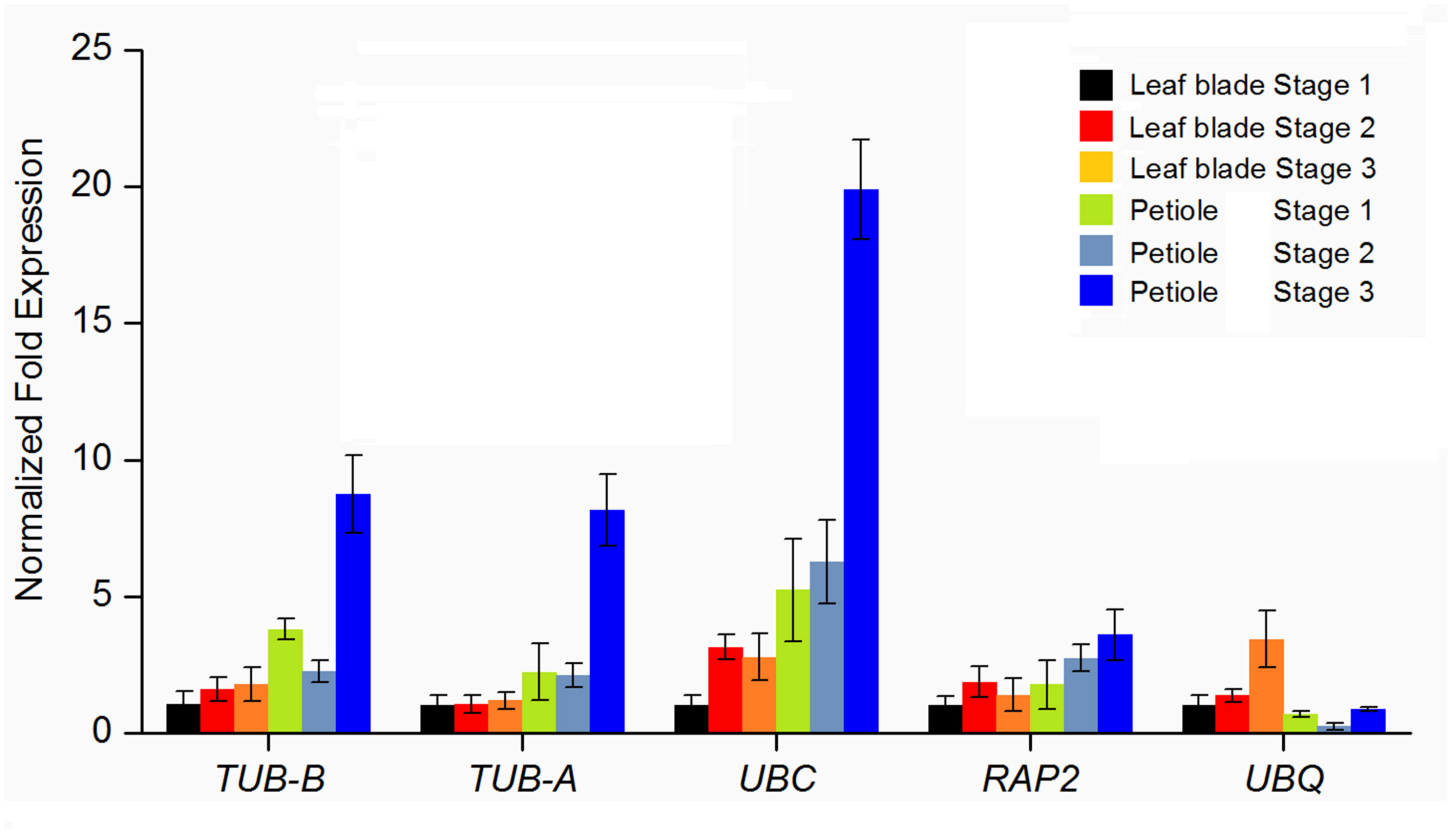
**Impact of different reference genes used for normalization on the relative quantification of ***AgAP2-2*** during leaf blade and petiole development**. *TUB-B, TUB-A, UBC, RAP2*, and *UBQ* were used as reference gene for expression normalization.

To validate the suitability of reference genes, five reference genes including two most stable reference genes *TUB-B* and *TUB-A*, two less stable reference genes *UBC* and *RAP2*, and the least stable reference gene *UBQ* were selected as calibrator. The relative expression levels of *AgAP2-2* were, respectively, calculated by using the selected reference genes. The homologous gene in *Arabidopsis, AtAP2* (AT4G36920), which have been confirmed to play an important role in plant development (Jofuku et al., 1994; Liu et al., 2014). In our transcriptome data (Jia et al., 2015), the transcript abundances of *AgAP2-2* were significantly different at three developmental stages. As illustrated in Figure 5, the expression patterns of *AgAP2-2* had a greater difference using different reference genes. When using the most stable reference genes *TUB-B* and *TUB-A*, the expression patterns of *AgAP2-2* were consistent, and the expression level was higher in petiole, especially at Stage 3. Similar expression patterns were generated by using the less stable reference genes *UBC* and *RAP2*. However, when the least stable reference gene *UBQ* was used, the expression level of *AgAP2-*2 had a strong bias compared with other genes. This result demonstrated that the reference gene with stable expression was necessary to accurately normalize the expression of target gene.

### Expression abundances of development-related genes in three development stages

Many genes have been identified to play key roles in plant growth and development, such as transcription factor genes, hormone-related genes, and genes encoding ribosomal proteins (Katagiri, 1992; Horiguchi et al., 2012; Saini et al., 2013). In *Arabidopsis*, lots of genes were identified to involve in development and multiple biological processes (Jofuku et al., 1994; Horiguchi et al., 2011; Köllmer et al., 2011). The high-throughput transcriptome sequencing of three celery development stages were finished by our lab and the annotation of celery genes showed many genes were involved in plant development (Jia et al., 2015). Basing on the transcriptome data in celery and the orthologous genes between celery and *Arabidopsis*, we selected 15 genes which, respectively, belonged to AP2/ERF transcription factor family, auxin-related gene, and ribosomal protein gene for further study. The expression abundances of these genes were analyzed by calculating the RPKM-values.

**Figure 6:**
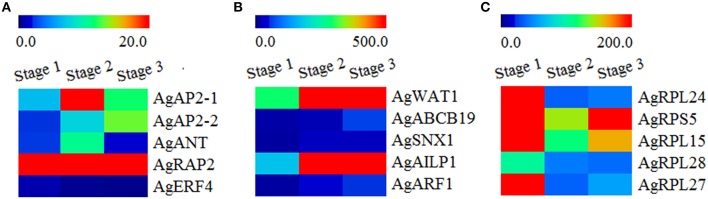
**Heatmap clustering of development-related genes expression abundances in three development stages. (A)** Transcript abundances of AP2/ERF family genes. **(B)** Transcript abundances of auxin-related genes. **(C)** Transcript abundances of ribosomal protein genes. RPKM-values were log_2_-based. Red and blue indicate high and low expression levels, respectively.

As showed in Figure 6, the auxin-related genes (*AgWAT1, AgABCB19, AgSNX1, AgAILP1, AgARF1*) and ribosomal protein genes (*AgRPL24, AgRPS5, AgRPL15, AgRPL28, AgRPL27*) have a relatively high abundance, whereas the AP2/ERF family genes (*AgAP2-1, AgAP2-2*, and *AgANT* belong to AP2 subfamily, *AgRAP2* belongs to RAV subfamily, *AgERF-4* belongs to ERF subfamily) were relatively low. Several genes which belonged to the same group showed a similar expression pattern, such as *AgWAT1*/*AgAILP1, AgABCD19*/*AgARF1*, and *AgRPL24*/*AgRPL27*. In addition, we also found that many genes showed differential expression at three development stages. Most AP2/ERF transcription factor genes and auxin-related genes showed low expression abundance at Stage1 and high abundance at Stages 2 and 3. It seemed that the expression of these genes increased over the process of plant development. In contract, the expression levels of all the ribosomal protein genes at Stage 1 were significantly higher than those at Stages 2 and 3.

### qPCR analysis of different expression genes in three development stages

**Figure 7:**
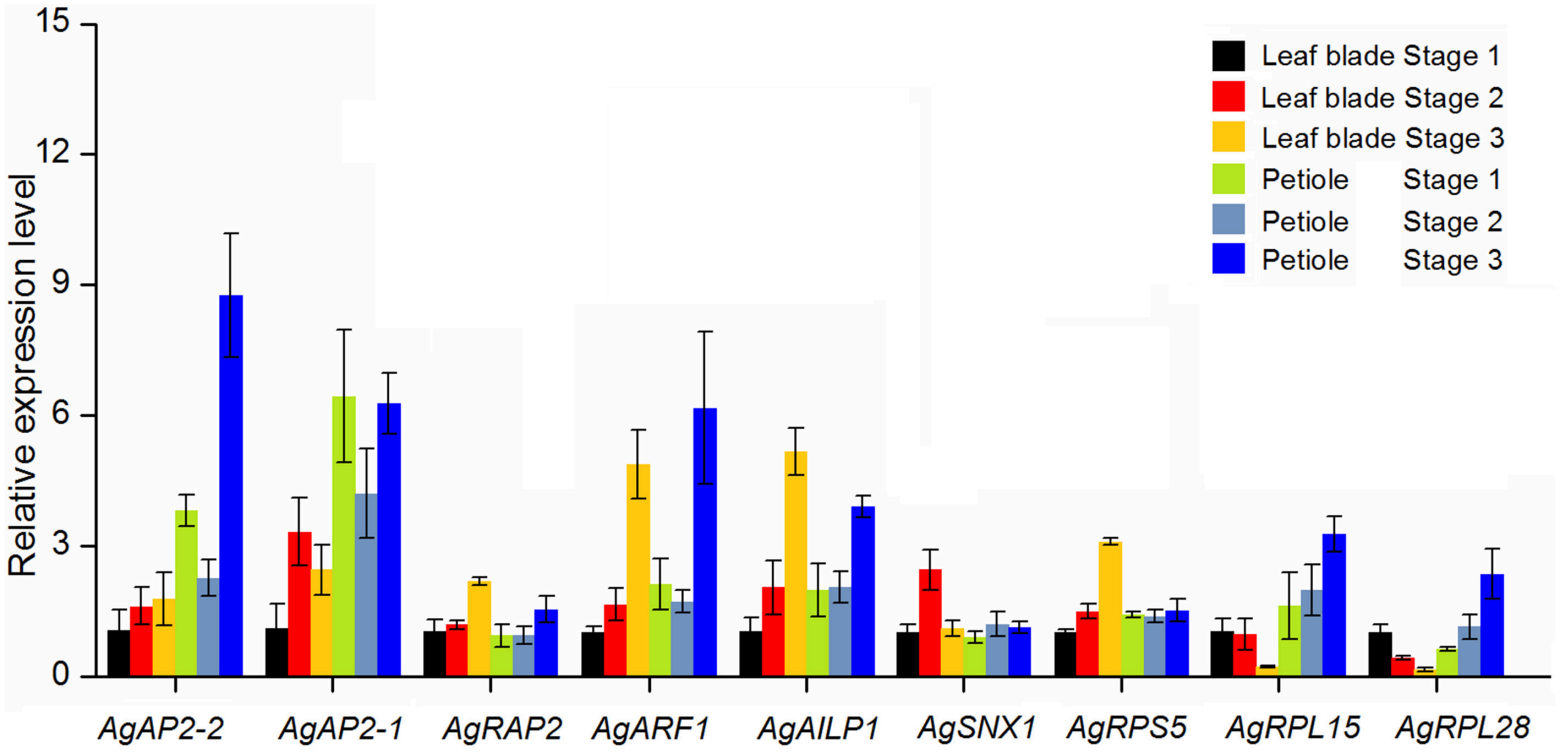
**qPCR analysis of the different expression genes in celery leaves blade and petioles at different developmental stages**. *TUB-B* was used as reference gene for expression normalization. The data are presented as the mean ± *SD* of three biological and technical replicates.

Among the selected genes, nine different expression genes were further selected to examine their expression levels in leaf blade and petiole at three development stages by qPCR (Figure 7). *TUB-B* was used as control gene to normalize the expression. The expression patterns of *AgAP2-1* and *AgAP2-2* were similar: the expression level was higher in petiole, especially at Stage 3. Overall, the expression of these two genes increased with the plant development. *AgRAP2* had a little change in leave blade and petiole at three stages, yet the expression abundance kept a high level in Figure 5. The two auxin-related genes, *AgARF1* and *AgAILP1*, also showed the similar expression pattern: the expression differences were not significant between Stages 1 and 2, but at Stage 3 the expression level increased about five times both in leaf and petiole. The other gene *AgSNX1* seemed to have a stable expression. For the three ribosomal protein genes, the expression level of *AgRPS5* in leaf blade had a slight increase, while in petiole showed no remarkable difference. Transcriptome data showed that this gene was detected with high abundance in three development stages (Figure 5). Interesting, the expression patterns of *AgRPL15* and *AgRPL28* in leaf blade were significantly decreased during the development of the leave blades, while the expressions in petiole were gradually increased.

## Discussion

qPCR has become an important tool for molecular biology research. Using a suitable reference gene can efficiently correct the errors of RNA quantity and reverse transcription efficiency, which can help to obtain the real differential expression of target gene (Udvardi et al., 2008). The most commonly used reference genes, which used as basic component of organelles skeleton (*ACTIN, TUB-A*, and *TUB-B*) or involved in biochemical metabolic processes of organisms (*GAPDH, EF-1*α, and *UBQ*), can stably express in tissues and organs (Huggett et al., 2005; Gutierrez et al., 2008). However, recent studies have found that these reference genes may show instability under various plant species or genotypes (Wang et al., 2014, 2015). For example, *GAPDH* showed the most stability in grapevine but ranked worst in wheat (Reid et al., 2006; Long et al., 2010); *UBI* and *ACT* showed good stability in wheat, yet performed unsatisfactory in tomato (Long et al., 2010; Mascia et al., 2010). Moreover, the expression stabilities of reference genes have been demonstrated to vary under different tissues and environmental stresses (Czechowski et al., 2005; Libault et al., 2008).

Since a large sequence data have been obtained in celery, the expression patterns and function analysis of many genes will be more convenient. As the vegetative organs, leaf blades and petioles are the product of specific development stage of celery. Previous study have revealed several key genes contribute to the complex network of celery development (Jia et al., 2015), further study involving the expression patterns and function analysis of these genes in various tissues and tissues developmental stages will be needed. To ensure accurate and reliable results, reference genes should be evaluated in target conditions. To date, there is no report on the selection of the most suitable reference genes in celery.

Three commonly used algorithms (geNorm, NormFinder, and BestKeeper) were used to evaluate and identify suitable reference genes (Vandesompele et al., 2002; Andersen, 2004; Pfaffl et al., 2004). In our study, geNorm ranked *TUB-B, TUB-A, UBC* as the best reference genes. NormFinder generated a similar ranking to the geNorm analysis. BestKeeper recommended *UBC, EF-1*α, and *UBQ* as the most stable reference genes under all samples. Taken all the results into consideration, *TUB-B, TUB-A*, and *UBC* can be used as reference for normalization in celery development. *ACTIN* is always considered as a suitable reference gene, but the results of the current study indicated that *ACTIN* is not the best suitable reference gene in celery. The *tubulin* gene family members, including *TUB-A* and *TUB-B*, also often serve as the suitable candidate reference genes. The expression stability of *TUB-A* is generally higher than the *TUB-B* (Brunner et al., 2004; Jian et al., 2008). But in this study, *TUB-B* appears to be more stable. Using a single reference gene for calibration and standardization is deemed to affect the accuracy of the result (Zhu et al., 2008). Schmid et al. (2003) suggested that two or more reference genes can contribute to the calibration of system deviation under a set of samples or experimental conditions. The geNorm programmer determined the optimal number of reference genes necessary for normalization under different samples in our study. With a threshold of 0.15, two reference genes were enough for normalization at Stages 1 and 2 in leaf blade and at Stage 3 in petiole, while more genes were needed for other tissue or conditions. In “Petiole” and “Total” groups, there was no suitable number of reference genes. The complexity of the samples may result in higher variability. However, using more reference genes can help to reach possible optimum results, but not a necessary criterion (Vandesompele et al., 2002).

A large number of genes are involved in the process of plant development. Some transcription factors, such as AP2/ERF, NAC, MADS-box, participated in cell differentiation, organ development, and construction of plant morphology (Rounsley et al., 1995; Köllmer et al., 2011; Le et al., 2011). In this study, genes encoding AP2/ERF family transcription factors expressed differently at different stages of celery development, suggested that these genes might involve in activity developmental regulation in celery. The expression levels of auxin-related genes and ribosomal protein genes were relatively high, especially the expressions of several ribosomal protein genes in early development stage. With the exuberant cell division activity, the young leaves require a lot of protein synthesis to meet the growing needs. The high expression of ribosomal protein genes in the initial stage of plant growth provides a prerequisite for translating other development-related genes. Some of the ribosomal protein genes associated with leaf development in *Arabidopsis* were also confirmed (Schippers and Mueller-Roeber, 2010). Overall, the coordinated regulation of a large number of development related genes has realized normal development of plants. Selection of the suitable reference genes provides a favorable basis for the further research on celery development.

## Author contributions

AX and ML conceived and designed the experiments. ML, GW, QJ, CT, and FW performed the experiments. ML, QJ, CT, and AX analyzed the data. AX contributed reagents/materials/analysis tools. ML wrote the paper. ML and AX revised the paper.

### Conflict of interest statement

The authors declare that the research was conducted in the absence of any commercial or financial relationships that could be construed as a potential conflict of interest.

## Supplementary material

Figure S1 **Standard curves of each candidate genes in leaf blades**.

Figure S2 **Standard curves of each candidate genes in petiole**.

Table S1 **Raw Cq-values of candidate reference genes**.

Table S2 **Oligonucleotide primer sequences**.

Table S3 **Data statistics of Cq-values of candidate reference genes in all samples**.

## Abbreviations

*ACTIN*: actin gene
*EF-1α*: elongation factor-1*α* gene
*GAPDH*: glyceraldehyde-3-phosphate dehydrogenase gene
*RAP2*: ethylene response factor RAP2 gene
*TBP*: TATA-box binding protein gene
*TUB-A*: α-tubulin gene
*UBC*: ubiquitin C gene
*TUB-B*: *β-tubulin gene*
*UBQ*: polyubiquitin gene
qPCR: quantitative real-time reverse transcription PCR.
