# Supplementary figures and images for: Validation and Comparison of Reference Genes for qPCR Normalization of Celery (Apium graveolens) at Different Development Stages
Source: Front Plant Sci. 2016 Mar 17;7:313. doi: 10.3389/fpls.2016.00313 (PMC4794502; doi:10.3389/fpls.2016.00313)

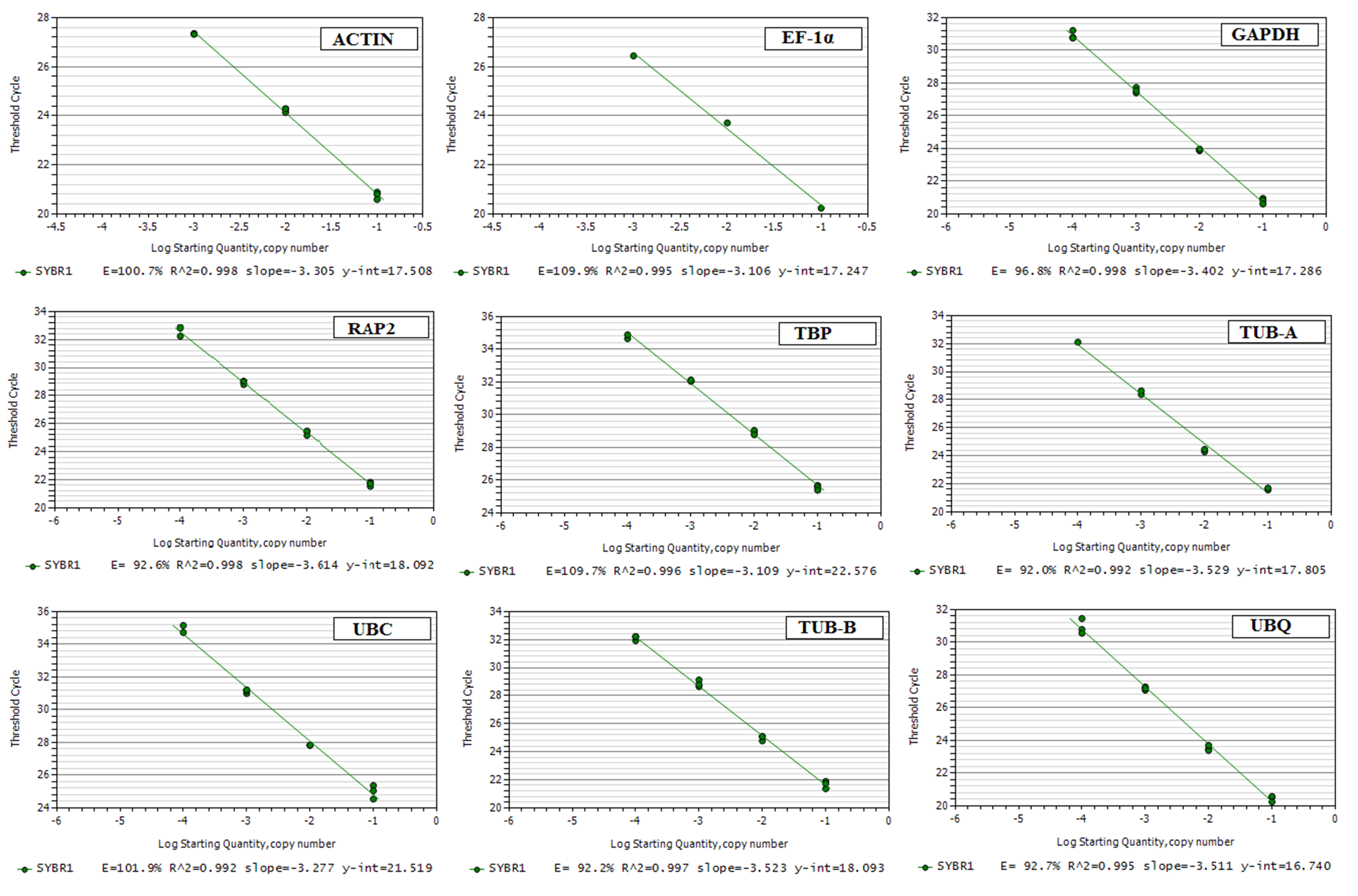

Supplement: Figure S1 — Standard curves of each candidate genes in leaf blades. [file Image1.TIF]

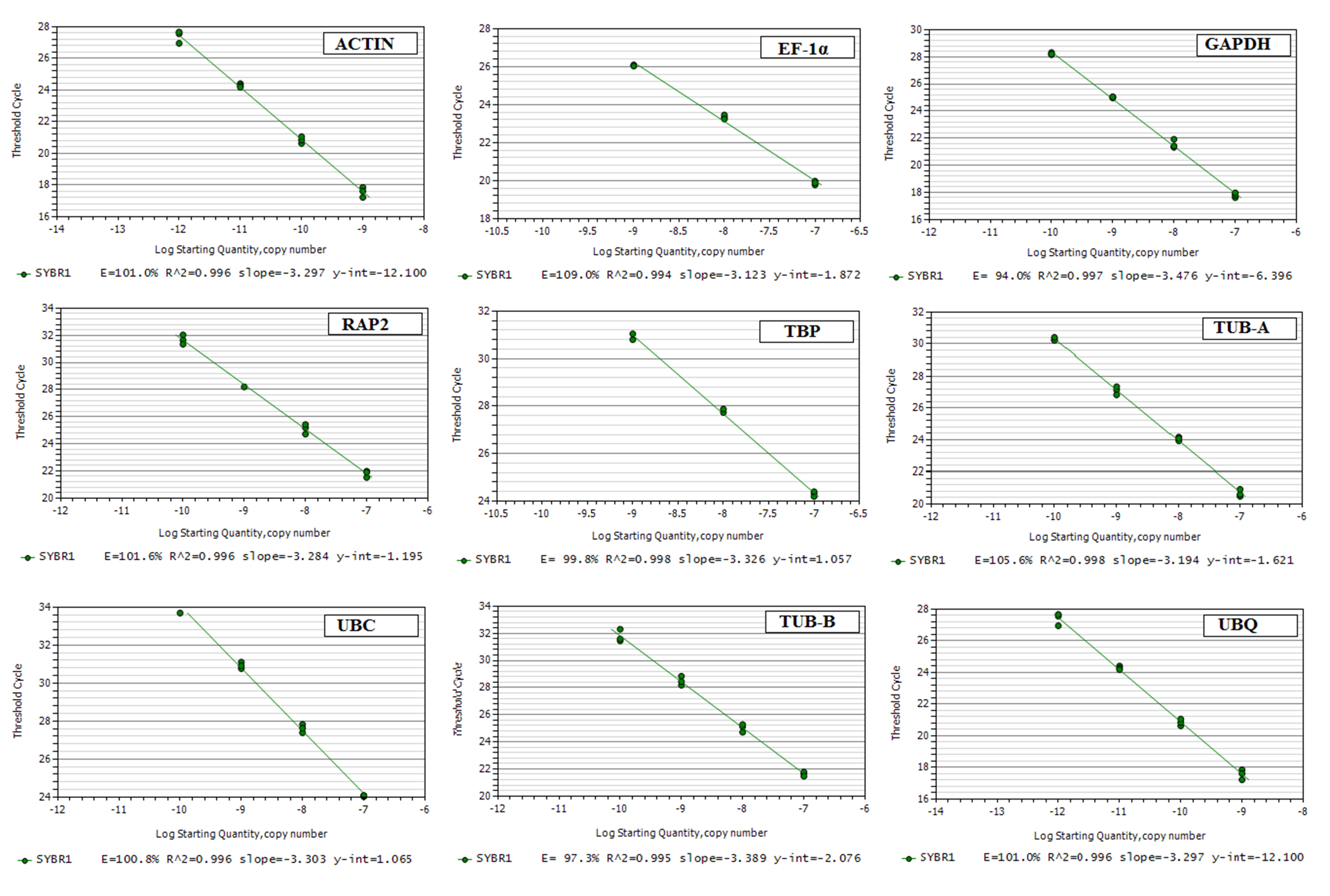

Supplement: Figure S2 — Standard curves of each candidate genes in petiole. [file Image2.TIF]
