# Supplementary material for: Validation and Comparison of Reference Genes for qPCR Normalization of Celery (Apium graveolens) at Different Development Stages
Source: Front Plant Sci. 2016 Mar 17;7:313. doi: 10.3389/fpls.2016.00313 (PMC4794502; doi:10.3389/fpls.2016.00313)
Supplement: Table S1 — Raw Cq-values of candidate reference genes. [file DataSheet1.docx]

Table S1 Raw Cq values of candidate reference genes.

| **Group** | Repeat | ACTIN | EF-1α | GAPDH | RAP2 | TBP | TUB-A | UBC | TUB-B | UBQ |
| --- | --- | --- | --- | --- | --- | --- | --- | --- | --- | --- |
| Stage 1  Leaf blade | R1 | 22.05 | 22.09 | 21.62 | 22.77 | 26.67 | 20.97 | 25.64 | 22.57 | 24.49 |
|  | R2 | 21.80 | 21.24 | 21.19 | 22.52 | 26.3 | 21.11 | 24.48 | 22.91 | 22.39 |
|  | R3 | 21.45 | 21.4 | 21.24 | 22.38 | 26.13 | 20.86 | 24.7 | 22.76 | 22.37 |
|  | R4 | 22.44 | 22.5 | 21.17 | 22.16 | 26.08 | 20.46 | 25.17 | 21.84 | 21.2 |
|  | R5 | 23.66 | 22.33 | 20.83 | 22.92 | 25.27 | 20.63 | 24.97 | 22.36 | 20.95 |
|  | R6 | 23.89 | 22.62 | 20.58 | 23.95 | 25.69 | 20.15 | 24.62 | 23.46 | 20.24 |
|  | R7 | 22.28 | 24.35 | 22.21 | 24.12 | 27.21 | 21.57 | 25.53 | 23.01 | 21.59 |
|  | R8 | 22.89 | 24.08 | 21.62 | 22.81 | 26.98 | 20.3 | 25.72 | 22.49 | 22.69 |
|  | R9 | 22.83 | 22.9 | 21.64 | 22.93 | 27.21 | 20.83 | 25.14 | 22.22 | 21.96 |
| Stage 2  Leaf blade | R1 | 25.75 | 25.53 | 23.23 | 24.88 | 28.39 | 20.89 | 25.38 | 24.71 | 22 |
|  | R2 | 25.92 | 25.25 | 22.95 | 24.64 | 28.29 | 20.92 | 25.31 | 24.04 | 22.09 |
|  | R3 | 26.46 | 25.7 | 22.83 | 25.74 | 28.33 | 21.72 | 25.99 | 25.24 | 22.47 |
|  | R4 | 25.07 | 23.98 | 24.8 | 25.27 | 26.98 | 23.58 | 27.15 | 25.53 | 20.68 |
|  | R5 | 25.16 | 23.87 | 24.49 | 25.11 | 27.69 | 23.66 | 27.13 | 25.61 | 21.3 |
|  | R6 | 26.09 | 24.2 | 24.95 | 25.89 | 27.21 | 24.65 | 26.93 | 25.92 | 21.3 |
|  | R7 | 26.41 | 25.56 | 24.09 | 25.73 | 28.83 | 23.6 | 26.97 | 25.2 | 23.83 |
|  | R8 | 27.33 | 25.34 | 23.97 | 25.13 | 27.82 | 23.94 | 26.6 | 25.15 | 23.68 |
|  | R9 | 26.90 | 25.32 | 23.86 | 24.66 | 27.08 | 22.64 | 26.42 | 25.44 | 23.65 |
| Stage 3  Leaf blade | R1 | 25.78 | 25.57 | 25.29 | 26.62 | 29.75 | 23.52 | 26.37 | 26.06 | 25.94 |
|  | R2 | 27.11 | 25.9 | 24.87 | 25.57 | 29.68 | 23.38 | 26.85 | 25.88 | 25.6 |
|  | R3 | 27.89 | 25.74 | 25.08 | 27.52 | 29.84 | 25.72 | 26.75 | 27.2 | 25.2 |
|  | R4 | 25.80 | 25.68 | 25.84 | 26.14 | 28.73 | 24.09 | 27.46 | 25.04 | 22.48 |
|  | R5 | 25.49 | 25.11 | 25.89 | 25.73 | 28.24 | 24.54 | 26.73 | 25.64 | 22.75 |
|  | R6 | 26.20 | 25.49 | 24.75 | 24.35 | 27.94 | 23.96 | 26.82 | 24.32 | 22.36 |
|  | R7 | 25.82 | 25.43 | 25.88 | 26.19 | 27.54 | 23.42 | 26.82 | 25.78 | 23.61 |
|  | R8 | 27.66 | 25.61 | 25.92 | 25.74 | 27.82 | 23.15 | 26.56 | 25.9 | 23.69 |
|  | R9 | 26.83 | 25.02 | 25.65 | 25.72 | 26.63 | 22.93 | 26.37 | 25.8 | 23.35 |
| Stage 1  Petiole | R1 | 24.78 | 23.53 | 17.62 | 24.32 | 26.55 | 18.51 | 22.01 | 21.12 | 22.15 |
|  | R2 | 25.33 | 24.38 | 17.25 | 24.85 | 25.11 | 18.76 | 23.42 | 19.7 | 22.47 |
|  | R3 | 24.72 | 24.63 | 17.03 | 24.46 | 25.25 | 19.11 | 23.22 | 21.24 | 22.45 |
|  | R4 | 20.93 | 21.63 | 19.09 | 22.08 | 23.92 | 19.04 | 23.93 | 21.65 | 22.62 |
|  | R5 | 21.61 | 21.26 | 18.75 | 21.77 | 23.82 | 18.89 | 23.27 | 21.02 | 22.60 |
|  | R6 | 21.47 | 21.34 | 18.71 | 22.08 | 23.82 | 19.18 | 23.66 | 21.32 | 23.16 |
|  | R7 | 21.97 | 22.48 | 19.54 | 23.61 | 25.60 | 20.36 | 24.04 | 22.08 | 22.61 |
|  | R8 | 22.72 | 24.60 | 19.21 | 22.74 | 24.81 | 20.99 | 23.65 | 21.31 | 22.97 |
|  | R9 | 22.37 | 23.44 | 19.18 | 23.78 | 25.88 | 21.04 | 24.33 | 21.69 | 22.60 |
| Stage 2  Petiole | R1 | 23.66 | 24.31 | 18.75 | 23.5 | 26.10 | 19.81 | 23.93 | 21.61 | 21.68 |
|  | R2 | 23.64 | 24.29 | 18.42 | 23.67 | 23.15 | 20.22 | 23.48 | 21.36 | 21.50 |
|  | R3 | 23.18 | 23.38 | 17.85 | 24.09 | 23.89 | 19.62 | 23.45 | 21.61 | 21.46 |
|  | R4 | 23.43 | 24.99 | 19.88 | 22.17 | 23.34 | 20.48 | 23.76 | 23.06 | 24.05 |
|  | R5 | 24.93 | 24.25 | 19.23 | 21.97 | 22.93 | 20.39 | 23.24 | 23.1 | 23.89 |
|  | R6 | 24.75 | 24.11 | 17.84 | 21.61 | 22.97 | 19.25 | 22.21 | 22.06 | 23.76 |
|  | R7 | 23.81 | 23.58 | 19.40 | 22.11 | 25.31 | 21.15 | 25.66 | 22.79 | 25.01 |
|  | R8 | 23.3 | 23.41 | 19.08 | 22.48 | 24.79 | 21.64 | 25.24 | 22.79 | 24.98 |
|  | R9 | 22.02 | 23.66 | 19.00 | 23.19 | 23.72 | 20.57 | 24.04 | 22.07 | 24.79 |
| Stage 3  Petiole | R1 | 24.94 | 25.26 | 21.58 | 26.37 | 25.31 | 21.38 | 25.22 | 22.77 | 23.64 |
|  | R2 | 25.17 | 26.18 | 21.72 | 26.73 | 25.66 | 21.29 | 23.01 | 22.12 | 23.34 |
|  | R3 | 25.27 | 26.73 | 21.52 | 26.78 | 24.83 | 22.24 | 24.49 | 22.42 | 24.02 |
|  | R4 | 23.39 | 25.47 | 21.58 | 24.26 | 25.18 | 22.29 | 24.68 | 23.34 | 24.91 |
|  | R5 | 24.05 | 25.87 | 22.25 | 25.65 | 25.63 | 21.93 | 25.22 | 23.68 | 25.64 |
|  | R6 | 23.23 | 25.37 | 21.92 | 25.2 | 24.79 | 22.17 | 24.62 | 23.91 | 24.95 |
|  | R7 | 22.50 | 25.46 | 22.31 | 25.33 | 25.77 | 22.35 | 24.88 | 23.63 | 25.47 |
|  | R8 | 22.88 | 25.06 | 22.23 | 25.36 | 25.74 | 22.59 | 25.06 | 23.52 | 25.32 |
|  | R9 | 22.68 | 25.28 | 22.23 | 25.15 | 25.47 | 22.41 | 24.9 | 23.89 | 25.28 |

Table S2 Oligonucleotide primer sequences.

| Gene | Primer sequence (5’-3’) | |
| --- | --- | --- |
| AgAP2-2 | | GAAGGTGGTAGTGCTGCTGGAA / CCCAAATATGCGACTCCCAACGA |
| AgAP2-1 | | TCTCACACCACTCCTAATGCTCCT / TCCTCGCCTGCTCTTGTTCAAC |
| AgRAP2 | | GAAGGTGGTAGTGCTGCTGGAA / CCCAAATATGCGACTCCCAACGA |
| AgARF1 | | CTCACAGCCTCAGACACAAGCA / GCCTCCTCACACCAACACGTAG |
| AgAILP1 | | CTGATGGCTACGTTGCGATTGC/ CAGTGGCAGGAACAGCAGTGAT |
| AgSNX1 | | CGCCTAATGGGTGAGGAGATTGT/ AGACTTCGCCAAGCATCAGCAA |
| AgRPS5 | | TGGCAGTTCGCATCATCAAGCA/ CTCACCACACCAGCAGAACCAA |
| AgRPL15 | | GGTTGATGCTGCCCACACTACT/ GTGATTGCGGTGACCCTTTCCA |
| AgRPL28 | | ACCGTATGGCAAAGGCAGTTTCT/ AAGGCTTCTGTTGACAGCACTCA |

Table S3 Data statistics of Cq values of candidate reference genes in all samples.

|  | N total | Median | Mean | SD | Minimum | Maximum |
| --- | --- | --- | --- | --- | --- | --- |
| *ACTIN* | 54 | 23.97 | 24.25 | 1.59 | 20.93 | 27.89 |
| *EF-1α* | 54 | 24.49 | 24.29 | 1.16 | 21.24 | 26.73 |
| *GAPDH* | 54 | 21.62 | 21.66 | 2.14 | 17.03 | 25.92 |
| *RAP2* | 54 | 24.41 | 24.31 | 1.33 | 21.61 | 27.52 |
| *TBP* | 54 | 25.98 | 26.18 | 1.46 | 22.93 | 29.84 |
| *TUB-A* | 54 | 21.22 | 21.57 | 1.42 | 18.51 | 25.72 |
| *UBC* | 54 | 25.10 | 25.06 | 1.13 | 22.01 | 27.46 |
| *TUB-B* | 54 | 23.04 | 23.39 | 1.45 | 19.70 | 27.20 |
| *UBQ* | 54 | 22.86 | 23.17 | 1.22 | 20.24 | 25.94 |
